# Psychosocial assessment of families caring for a child with acute lymphoblastic leukemia, epilepsy or asthma: Psychosocial risk as network of interacting symptoms

**DOI:** 10.1371/journal.pone.0230194

**Published:** 2020-03-23

**Authors:** Chiara Colliva, Monica Cellini, Francesca Dalla Porta, Martina Ferrari, Barbara Maria Bergamini, Azzurra Guerra, Silvia Di Giuseppe, Annamaria Pinto, Roberto Capasso, Daniela Caprino, Marta Ferrari, Cristina Benatti, Fabio Tascedda, Johanna M. C. Blom

**Affiliations:** 1 Department of Biomedical, Metabolic and Neural Sciences, University of Modena and Reggio Emilia, Modena, Italy; 2 Department of Medical and Surgical Sciences, Division of Pediatric Hemato-Oncology, University Hospital Azienda Policlinico di Modena, Modena, Italy; 3 Department of Medical and Surgical Sciences, Division of Pediatric Pneumology, University Hospital Azienda Policlinico di Modena, Modena, Italy; 4 Department of Medical and Surgical Sciences, Division of Pediatric Neurology, University Hospital Azienda Policlinico di Modena, Modena, Italy; 5 Center for Pediatric Hemato-oncology, University Politecnica delle Marche, Ancona, Italy; 6 Department of Oncology, Hospital Santobono Pausilipon, Naples, Italy; 7 Pediatric Hematology and Oncology, Institute Giannina Gaslini, Genova, Italy; 8 Department of Life Sciences, University of Modena and Reggio Emilia, Modena, Italy; 9 Center for Neuroscience and Neurotechnology, University of Modena and Reggio Emilia, Modena, Italy; Fordham University, UNITED STATES

## Abstract

The purpose of this study is to assess psychosocial risk across several pediatric medical conditions and test the hypothesis that different severe or chronic pediatric illnesses are characterized by disease specific enhanced psychosocial risk and that risk is driven by disease specific connectivity and interdependencies among various domains of psychosocial function using the Psychosocial Assessment Tool (PAT). In a multicenter prospective cohort study of 195 patients, aged 5–12, 90 diagnosed with acute lymphoblastic leukemia (ALL), 42 with epilepsy and 63 with asthma, parents completed the PAT2.0 or the PAT2.0 generic version. Multivariate analysis was performed with disease as factor and age as covariate. Graph theory and network analysis was employed to study the connectivity and interdependencies among subscales of the PAT while data-driven cluster analysis was used to test whether common patterns of risk exist among the various diseases. Using a network modelling approach analysis, we observed unique patterns of interconnected domains of psychosocial factors. Each pathology was characterized by different interdependencies among the most central and most connected domains. Furthermore, data-driven cluster analysis resulted in two clusters: patients with ALL (89%) mostly belonged to cluster 1, while patients with epilepsy and asthma belonged primarily to cluster 2 (83% and 82% respectively). In sum, implementing a network approach improves our comprehension concerning the character of the problems central to the development of psychosocial difficulties. Therapy directed at problems related to the most central domain(s) constitutes the more rational one because such an approach will inevitably carry over to other domains that depend on the more central function.

## Introduction

Pediatric chronic or severe illness is often accompanied by the development of significant psychosocial challenges that influence the present as well as the future burden of disease [1–3]. While overall, families of children with severe or chronic pediatric illness adjust well, there is growing evidence that some may develop significant difficulties at the psychosocial, cognitive, emotional and behavioral level [4–7]. Despite their mostly intact global intellectual functioning, children with epilepsy often display difficulties in behavior and domains of executive function, such as, working memory, attention, and planification which negatively affect academic performance [8–10] and emotional outcomes with a higher risk for emotional disorders such as anxiety and depression [11,12] including post-traumatic stress disorder (PTSD) [13].

Although often underestimated, children with asthma, not only display cognitive impairment, with a higher incidence of difficulties in attention related processes, but they also display a higher prevalence of anxiety and mood related disorders as compared to children with other chronic illnesses or to healthy children [14–16].

Recently, several studies have introduced the use of network analysis to highlight the different cognitive, emotional, and behavioral patterns that are present in children with different pediatric conditions, accentuating that patients’ and family characteristics are not simply the sum of separate abilities but the result of complex dynamic interactions [17–19]. Studies conducted across a variety of the most prevalent and serious pediatric illnesses, such as, acute lymphoblastic leukemia (ALL), epilepsy, and asthma have identified a range of potential adverse consequences including pressure on social support, negative impact on parent psychological health, family relationships and sibling well-being, as well as a direct psychological influence on the ill child [20–24]. In addition, the treatment of asthma, epilepsy, and ALL involves challenging medical regimens, in which families are confronted with multiple and often pervasive stressors including significant medical side effects [25–29] that considerably interfere with body function, cognitive and behavioral processes and daily activities [30,31]. Families must frequently make substantive changes in their structure and function, involving a redistribution of roles and responsibilities, ensuring financial stability, and providing a caring environment while monitoring their child’s illness and treatment. Consequently, the duration and intensity of the illness or treatment, as well as redefining rhythms and routines, enhance the risk for the onset of psychiatric diseases and reduced quality of life over time [32–34]. Moreover, the traumatic effects of prolonged and intense stress may become stratified and lead to the onset of disorders such as PTSD both in children as in their parents [35–37] with important costs in terms of emotional, financial, and family resources [38].

Furthermore, when left untreated, impaired psychosocial function may intensify with time, becoming more pronounced as children attempt to master more complex tasks that require the integration of multiple social and cognitive domains [2,33,39]. Consequently, one of the main challenges when caring for a chronically or severely ill child is to understand the role of various functional domains [40], such as, the structure and economic situation of the family, the stress reactivity of the caregiver, family beliefs, and patient related problems [41–44]. More importantly, however, is the relationships and connectivity among these domains [45–47].

Innovative discoveries from other fields, such as, imaging techniques and mathematical modeling together with graph analysis have led to new conceptual thinking resulting in increasingly explanatory and predictive models which may offer a more realistic image of the psychosocial strengths and needs of the families of these patients [48–51]. New imaging techniques that link cognition, behavior, and emotion to dynamic alterations in brain structure and function [52,53] have presented important new challenges because of the need to integrate an enormous amount of data [54]. This resulted in a growing necessity for more sophisticated and systemic methods to facilitate the analysis of the intrinsic properties of the relationships and dynamic interdependencies among the many variables and data collected. A new language that helped to quantify and describe the nature of the data was provided by the field of mathematics [55], used, at first, in ecology, epidemiology and sociology, to analyze the complex interdependencies between structure and function, allowing to predict the spread of a disease, the effect of the environment, or the hypothesized impact of therapeutic interventions [56,57].More specifically, the mathematical discipline underlying the study of the formation and dynamic character of complex networks is called graph theory [56,58,59]. The application of network analysis and graph theory to fundamental questions in neuroscience and to the interaction and nature of social groups formed the emerging fields of network neuroscience and social network theory [60,61]. By considering social groups as a system of interconnected points, called nodes, social network theory underscores the possibility for emergent properties to occur as a result of the intricate patterns of relationships between these points or individuals (called edges) while each contributing factor in isolation would not uncover these emergent properties. Moreover, many social environmental risk factors affect psychosocial stress and have been found to involve neural circuits whose structural and functional organization often experiences permanent and pervasive change in response to lengthy exposure [62]. The inherent relational nature of (social) network theory allowed researchers to study the relationships among individuals, family, groups, and even society, stressing the dynamic nature of the relationships and connections among nodes while giving less weight to the individual. Thus, network analysis may provide a universal way to study prevention, spread, and treatment, as well as the structure and psychosocial risk related to health and illness [45,63]. In particular, network analysis may help to improve our comprehension concerning the character of the problems and the domains central to the development of psychosocial difficulties which is fundamental for surveillance and monitoring vulnerability over time [49,50,64,65]. Therefore, to gain a better understanding of the functionality of the psychosocial context surrounding the ill child, we considered psychosocial risk as a network of interconnected and interdependent domains and explored the hypothesis that different illnesses are characterized by different network profiles of needs and risks. If so, this type of approach may have important implications for clinical practice as it not only indicates which domains are most compromised but also which domains are most central to the network and, therefore, may drive the overall psychosocial risk of the family [66,67]. Alternatively, we evaluated whether children with severe or chronic pediatric conditions may be characterized by clusters of problems in specific domains [66,68] that represent a common profile of psychosocial needs of the sick child in general. In sum, the purpose of this study, was to evaluate the differences and similarities of psychosocial risk in families caring for a child with ALL, epilepsy or asthma and how to best analyze the dynamic interdependencies among various domains of psychosocial risk. More explicitly, the objectives were: (1) To assess whether the overall level of psychosocial risk differs among various serious or chronic pediatric conditions; (2) To evaluate if domains of psychosocial functioning diversely affect families caring for a child diagnosed with ALL, epilepsy or asthma; (3) To establish the weight of individual psychosocial domains within the overall risk profile and their centrality to other psychosocial risk factors; and (4) To determine whether a common risk profile exists for families caring for a severely or chronically ill child. Answers to these questions may help to quickly and effectively identify the subset of children and families at greatest risk for ongoing and/or escalating psychosocial difficulties and implement psychosocial screening based on a network approach starting early after diagnosis in order to recognize interrelated behaviors that add to the risk and burden of children, adolescents and their families by isolating the domain(s) most central to overall psychosocial risk and implementing therapy accordingly.

## Materials and methods

### Participants

Families of 195 children, aged 5–12, were enrolled at the out-patient clinics and day-hospitals of participating centers, two centers were generalized pediatric hospitals with pediatric oncology, pneumology and neurology as operative units (Ancona and Genova) one hospital was a general hospital with all pediatric subspecialties (Modena), and one hospital was a pediatric oncology hospital (Napoli, SantoBono Pausilipon). Participants were parents of children diagnosed with asthma, epilepsy, and ALL admitted to or receiving care at one of the participating centers. Admission of the target patient groups was checked by the research team, in coordination with the clinical staff of each pediatric specialty unit (pediatric pneumology, pediatric neurology, pediatric oncology); parents of the eligible families were approached by a member of the clinical staff and a senior researcher and recruited during their child’s hospital stay or admission.

Ninety children were diagnosed with acute lymphoblastic leukemia (ALL), forty-two with epilepsy, and sixty-three with asthma. Diagnostic groups were selected based on their frequency and importance among the various chronic and/or severe pediatric illnesses and on the different types of stress they may generate in the patient and the families caring for the patient: (1) Asthma is the most common chronic disease in childhood, in which children with frequent clinically significant exacerbation of symptoms, perceive their illness as stressful with serious concerns for the family; (2) Epilepsy, the most frequent neurologic pediatric condition, a chronic disease, that often profoundly perturbs the family and the child as manifestations are acute, severe, and treatment may have important side effects causing an enhanced burden of disease.(3) Finally, ALL, the most frequent pediatric malignancy, an acute and severe, potentially life threatening condition characterized by a long and burdensome treatment protocol which profoundly affects the patient as well as the family. All participants met eligibility criteria for the study: (1) a confirmed diagnosed of asthma, according to the criteria of the Global Initiative for Asthma [69,70] (GINA 2018), of ALL according to the BFM-AIEOP protocols (2000–2009) and of epilepsy based on a neurologic work-up according to the International League Against Epilepsy guidelines [71,72]; (2) not being affected by comorbid conditions known to alter cognition; and (3) sufficient comprehension of the Italian language. Exclusion criteria were brain lesions or genetic and metabolic disorders that could justify the onset of epilepsy.

The Ethics Committee of Modena approved the protocol and written informed consent and age-appropriate assent (from 11 years on) was obtained from all young participants and their parents.

### Study design

Participants were enrolled in a prospective longitudinal observational multicenter study. Here, we report on the first time points. The objective of this observational study was to screen and identify families of severe pediatric medical conditions for unmet psychosocial needs and problems, using the Psychosocial Assessment Tool (PAT2.0) [73–75] and to better understand the interaction among various psychosocial risk factors.

### Procedures

The PAT was completed by parents during the first visit after enrollment in the study. The first visit of patients diagnosed with asthma and epilepsy coincided with the first regular visit envisaged by their treatment plan. For ALL, the first visit coincided with the early phase of treatment (1 to 2 months after diagnosis). Patients were at the same time frame for the second time point as we reassessed all patients one year later, a period during which psychosocial risk was included as an important variable in their treatment plan. Note, this study is part of a larger longitudinal study during which patients will be assessed yearly for five years. Here we report on the first two time points; At T0 parents enrolled by the clinicians completed the PAT 2.0 (time to complete, 10–15 minutes) and they repeated the questionnaire one year later (T1). Parents also completed a semi-structured anamnesis developed for this study. A protocol was developed for the distribution, collection and scoring of the PAT2.0 (ALL patients) and the generic version of the PAT (epilepsy and asthma patients) [76–81]. Based on the PAT risk level and informed by the families and patients special needs, participants were referred for consultation. On-site social service and assistance as well as specialized pediatric psychologists were available for families with significant social needs. Social assistance often consisted in offering transport or temporary housing of the family, providing “a home away from home”, to the parents. Psychological assistance ranged from individual to group therapy for both patients and parents. Demographic data included: child age and sex, ethnicity, literacy, and family structure (age; instruction; marital status, ect.). Information related to family structure was collected by PAT subscales and a semi structured anamnesis. Disease specific clinical data were obtained from medical records such as diagnosis, type of treatment protocol, presence of treatment related side effects, age of onset.

### Measures

**Psychosocial assessment tool 2.0.** Psychosocial risk was measured using the Psychosocial Assessment Tool (PAT), Italian version. The PAT2.0 and PAT 2.0 generic version **(**PAT2.0_GEN) [77,78,80,82–84] are brief (10–15 minutes) parent-reported screening tools of psychosocial risk in families with a child with cancer or with other severe or chronic illnesses based on an ecological approach. The generic version of the PAT is an adapted version of the PAT which was initially developed for the pediatric oncology setting and has been adjusted for other patient populations affected by inflammatory bowel disease, obesity, congenital heart disease, diabetes and kidney transplant [76,78,79,81,83,85–87]. The PAT2.0 and PAT2.0_GEN are composed of 15 item sets and yield a total score (possible range 0–7) and seven subscale scores (possible range of 0–1) that include: Family Structure and Resources (8 items), Family Social Support (4 items), Family Problems (8 items), Parent Stress Reactions (3 items), Family Beliefs (4 items), Child Problems (15 items) and Sibling Problems (15 items). Response formats differ with respect to subscale [73,77,78,87,88]. The PAT2.0_GEN only differs from the original PAT2.0 on three items which were removed: (items 9, 15g and 15i). The total PAT score ranges from 0 to 7 (Table 1) [88]. Subscale scores are derived by dividing the number of high-risk items observed by the total number of scored questions on the respective subscale. Each item is scored dichotomously (risk = 1; no risk = 0). The adjusted score for each subscale ranges from 0.00 to 1.00.

**Table 1:** Partition of PAT risk.

PAT TOTAL SCORE	RISK LEVEL	DESCRIPTION
<1	UNIVERSAL	Low psychosocial risk level. Families are generally competent and capable of coping and adapting to the illness and treatment demands with education and support.
1.0–1.9	TARGETED	Intermediate psychosocial risk level. Families are at elevated risk for developing difficulties in illness adjustment and/or family functioning. Acute problems or stressors may be present.
>2	CLINICAL	High psychosocial risk level. Families present multiple stressors and risks that may necessitate clinical and psychosocial resource utilization

### Network analysis and graph theory

The aim of this study was to analyze the interrelationship of psychosocial risk factors and social needs in three medical conditions rather than comparing the differences among them at any given point in time. Therefore, for each pathology a psychosocial network was constructed based on undirected weighted adjacency matrices of 38 nodes, whereby each node represented one item on the PAT. Weighted connections between the nodes represented the strength of the correlation of those nodes across all individuals in each group. Items on the optional Sibling Problem domain were not included in the network (which resulted in a total of 42 items minus 4 unscored items, that is 38 items instead of the 57 items of the total PAT) (Table 2). Subsequently, more restricted summary networks were created using the seven subdomains of the PAT to define the weight of each subdomain in driving the overall psychosocial risk. Graph thresholding was performed, and only the significant correlations between nodes were included in the summary network [17,89]. The Fruchterman Reingold algorithm (Gephi open source software (http://gephi.github.io/) [90–92] was used to represent the community structure of interrelationships among the various items and domains of function that compose the PAT (gravity = 10, and speed = 1.0). The circular constellation with less important nodes pushed towards the outer layer, facilitated comparison among networks of different medical conditions [50,51,93,94]. Networks analyses were performed with Specific Rgraph packages.

**Table 2:** Items on the PAT 2.0 and PAT generic version included in the network analysis.

Item	subscale	Label	Description of item
**1**	struct/resources	mag18	Fewer than 2 people older than 18 years old
**2**	struct/resources	educ	Caregiver’s highest education (check boxes)
**3**	struct/resources	statciv	Patient’s parents’/guardians’ relationship status
**4c**	social support	finsup	Who can provide financial support
**4d**	social support	supinf	Who can provide information
**4b**	social support	emotsup	Who can provide emotional support
**4°**	social support	childcare	Who can provide childcare/parenting
**5**	struct/resources	transport	Means of transportation
**7/8**	struct/resources	econdif	current economic difficulties
**11°**	child problems	pchangemood	Change moods quickly?
**11b**	child problems	pyounger	Act younger than his/her age?
**11c**	child problems	pupsetmd	Get upset about going to the doctor/dentist
**11d**	child problems	phyperactivy	Act overly active? (i.e. hyperactivity
**11e**	child problems	pattdiff	Have attention difficulties/ADHD
**11f**	child problems	pcry-upset	Cry easily or become upset easily?
**11g**	child problems	pdistracted	Seem easily distracted?
**11h**	child problems	pworry	Worry?
**11i**	child problems	plearndif	Have learning or school difficulties?
**11j**	child problems	psad	Appear sad or withdrawn
**11l**	child problems	pdevdelay	Have developmental concerns or delays?
**11m**	child problems	pshy	Act shy or cling to you/other familiar adults?
**11n**	child problems	ppeerdif	Have difficulty making and keeping friends?
**11p**	child problems	violence	Been a victim of violence?
**13°**	family problems	exec worry	Has anyone experienced periods of excessive worry, fear and/or anxiety
**13b**	family problems	subabuse	Has substance use ever caused problems for anyone in the family?
**13c**	family problems	saddness-depr	Has anyone experienced periods of prolonged sadness or depression?
**13d**	family problems	attentiondif	Does anyone have difficulty focusing, concentrating and/or have a history of an attention deficit disorder?
**13e**	family problems	maritaldif	Have there been marital difficulties, conflict or discussion of separation?
**13g**	family problems	alcoholprob	Has anyone ever been told that s/he drinks too much?
**13h**	family problems	childcustody	Have there been any difficulties with child custody disputes?
**13j**	family problems	psychprob	Any other psychological conditions
**14°**	caregiver stress	nightmares	Have you had bad dreams or nightmares about your child being ill?
**14b**	caregiver stress	anxiety	Have you become jumpy since your child came to the hospital?
**14c**	caregiver stress	overly reactive	When you are reminded of your child being ill, do you sweat or tremble, or does your heart beat fast?
**15°**	family believes	faithmd	The doctors will know what to do
**15c**	family believes	faithfam	Our family will be closer because of this
**15f**	family believes	faithtreatment	We can make good treatment decisions
**15h**	family believes	faithoutcome	We’re going to beat this

#### Network centrality measures

Graph theory provides a measure of the architectural organization of psychosocial function, as defined by the network formed by the interrelationships between multiple psychosocial needs and domains. For this initial exploratory analysis, we used basic network centrality measures to test the fundamental role and influence of a node (item/symptom/domain) within the psychosocial network representative of disease specific needs. Centrality was measured according to degree-centrality, betweenness centrality, and closeness centrality [95]. Degree-centrality indicates the importance of a node in the network and quantifies the number of connections of an item/symptom/node. If a group of patients tend to develop a problem that is central, that is, the item or problem has many connections, the probability of developing other problems (connected to this problem) will increase more than when an item or problem has fewer connections [95]. Second, betweenness centrality (BC) was analyzed, which is a measure of the importance of a node in the communication of a network [40,96,97] because it is based on the number of shortest paths in which a node participates. Betweenness centrality shows which domains act as ‘bridges’ between other domains in the network. Here, we used it to determine which domain(s) is most important in influencing the connectivity among problems and symptoms which allowed us to analyze the dynamics of the psychosocial network characteristic of each pathology [18,40,98]. Finally, closeness centrality was used to understand the importance of a symptom or problem and its immediate influence on the function of neighboring nodes. The combinations of these three different centrality measures (i.e., degree and betweenness centrality) helped to identify the most important or “dominant” domain(s) for the configuration of our network and thus where to direct therapy and reach other domains most efficiently [94,98].

### Statistical analysis

SPSS Statistics 25.0 for (SPSS Inc., Chicago, Illinois) was used for all statistical analyses. Demographic and disease characteristics were compared among the families of three severe pediatric conditions. Group differences for all subscales were tested for sex and age using a univariate general linear model (GLM), with sex and age at testing entered as covariates. Linear regression analyses were conducted to further explore the predictive value of illness on each subscale of the PAT (dependent variables). Predictive effects of illness and sex were considered significant at the .05 level and are presented as odds ratios (OR) with 95% confidence intervals. Correlation coefficients were used to construct the network and perform graph analysis. A first network was constructed using all items included in the subscales (except for items on the sibling problem subscale). Second, we constructed summary networks for each condition. Subsequently, graphs were analyzed in a reduced form using statistical thresholding based on significant correlations (alpha level of p < .05) to illustrate the difference of the weight of each subscale within the network as well as the level of organization of the network. Finally, data-driven cluster analysis was conducted to test the alternative hypothesis that different conditions share domains of psychosocial risk.

## Results

### Differences between PAT total and PAT subscale scores among families caring for a child with ALL, epilepsy or asthma

PAT data were collected from 195 families across 4 sites. Table 3 represents the sociodemographic and clinical characteristics of the sample. Most children were male (65.8%) and Caucasian (43%). The total sample of the 3 conditions included 195 children (mean age 9.8 years SD 3.4) and their parents (mothers mean age, 42.4 ± 5.46 years; father mean age, 45.0 ± 6.3 years). Caregivers were mostly married or in a stable relationship (81.5%, n = 159).

**Table 3:** Demographic and clinical characteristics of children diagnosed with acute lymphoblastic leukemia, epilepsy and asthma (n = 195).

	Asthma n = 63	Epilepsy n = 42	ALL n = 90	Total sample n = 195
**CHILDREN’S CHARACTERISTICS**				
**age, years, mean ± SD**	10.1 ±2.8	8.8± 2.4	10.5 ± 5.6	9.8 ± 2.8
**age at diagnosis, years, mean ± SD**	4.2 ± 2.9	5.2± 2.9	6.3 ± 3.9	4.8 ± 3
**AGE GROUP, N (%)**				
**age < 7 years old**	11 (17.5)	14 (33.3)	8 (8.9)	33 (16.9)
**8 < age > 10 years old**	25 (39.7)	16 (39)	37 (41.1)	78 (40)
**age > 10,6 years old**	27 (42.9)	12 (28.6)	45 (50)	84 (43.1)
**GENDER, N (%)**				
**Male**	50 (79.4)	29 (69)	48 (53.3)	127 (65.1)
**Female**	13 (20.6)	13 (31)	42 (46.7)	68 (34.9)
**PARENT’S CHARACTERISTICS**				
**mother age, years, mean ± sd**	41.5 ± 6.4	41.2 ± 4.4	44.5 ± 5.6	41.7 ± 5.7
**father age, years, mean ± sd**	43.5 ± 5.9	44.1 ± 5.2	47.5 ± 7.7	45.1 ± 5.9
**MARITAL STATUS, N (%)**				
**single**	5 (7.9)	3 (7.1)	27 (30)	35 (17.9)
**married/stable relationship**	57 (90.5)	39 (92.9)	63 (70)	159 (81.6)
**widowed**	1 (1.6)	0	0	1 (0.5)

Children diagnosed with ALL were stratified according to protocol (BMF- AIEOP 2009). The sample of children with epilepsy was composed of 42 patients, of which 50% with BECT epilepsy and 12 patients (28.6%) with Temporal Lobe Epilepsy (TLE), the other 9 patients were classified as “other” with different diagnosis (frontal lobe epilepsy (n = 1), absence (n = 1), and various forms of epileptic dysphasia (n = 7)). Regarding the clinical characteristics of patients with asthma, 36.5% (n = 23) had mild persistent asthma, 42.9% (n = 27) moderate persistent asthma, and 15.9% had persistent severe asthma while only 4.8% (n = 3) had intermittent asthma.

**Fig 1:**
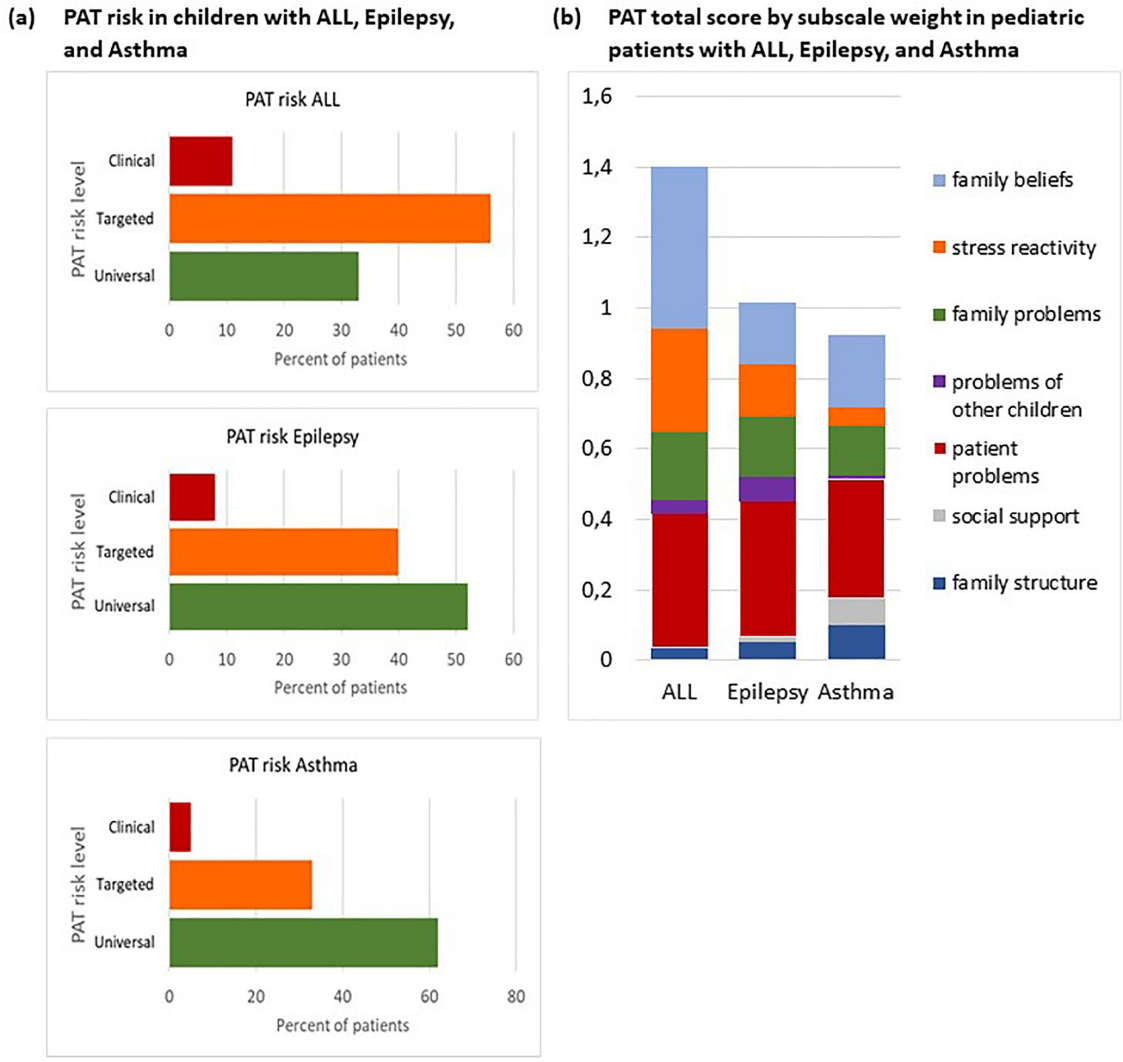
PAT risk, total score and subscale weight. a. PAT RISK in children with ALL, epilepsy, and asthma expressed as percentages at three levels of risk, universal, targeted, and clinical: b. Proportional weight of each subscale within the total PAT score.

The PAT separated the psychosocial risk of families into three levels (Fig 1A). Most families belonged to the universal level of psychosocial risk and only a minimal part of families scored in the clinical range (Fig 1A). Though generally in agreement with the data from the authors of the scale [81,88], few families caring for a child with a chronic illness, such as, epilepsy and asthma, displayed psychosocial risk (8% and 5% respectively) at the clinical level while most families (52% and 62% respectively) displayed universal psychosocial risk. Differently, more than 10% of families with a child diagnosed with ALL showed clinical psychosocial risk. Surprisingly, a substantial number of families, more than 55%, displayed an intermediate risk level and scored in the targeted range while only 33% scored in the universal range of the scale, displaying low psychosocial risk.

PAT total risk and PAT subscale distribution in pediatric ALL, epilepsy and asthma showed that in families caring for a child with ALL, the mean PAT score was 1.44 ± 0.48. Scores were highest in the domains of Family Beliefs (mean 0.46 ± 0.17) and Patient Problems (mean 0.37 ± 0.19), followed by Family Problems (mean 0.19 ± 0.13), Family Stress Reactivity (mean 0.29 ± 0.22), and Sibling Problems (mean 0.04 ± 0.13), while Social Support and Family Socioeconomics Structure and Resources were considered not to be problematic and did not add to the overall psychosocial risk (Fig 1B).

In families caring for a child with epilepsy or asthma, the mean PAT score was 1.0 ± 0.54 and 0.90 ± 0.6, respectively. Families caring for a child with epilepsy, scored highest in the domain of Patient Problems (mean 0.39 ± 0.20) and moderate in the domains Family Problems, Stress Reactivity, and Family Beliefs (mean 0.17 ± 0.14, mean 0.15 ± 0.27, and mean 0.17 ± 0.20, respectively). Family Structure (mean 0.05 ± 0.09) and Social Support (mean 0.02 ± 0.07) did not contribute in a major way to the total PAT score. In families caring for a child with asthma, Family Structure (mean 0.10 ± 0.16) and Social Support (mean 0.08 ± 0.18) together counted for almost twenty percent of the PAT score, which was significantly higher than in the other two conditions. Also, scores were highest in the domain of Patient Problems (mean 0.34 ± 0.20), followed by Family Beliefs (mean 0.20 ± 0.23) and Family problems (mean 0.14 ± 0.18), while Sibling Problems (mean 0.008 ± 0.26) and Stress Reactivity (mean 0.054 ± 0.16) were reported to be absent or almost absent. Overall, when controlled for sex, type of pathology was associated with different total PAT scores; psychosocial risk of families caring for a child with ALL was significantly higher than in families caring for a child with epilepsy or asthma (F (2,191) = 23.191, p< 0.001, ɳ2 0.131). No difference was observed in the total PAT score between the latter two. Also, type of pathology differentially affected the subscale scores of the PAT (F(14,366) = 17.141, *P* < 0.05, Wilks Ʌ = 0.696, partial η^2^ = 0.348), and influenced, Family Structure (F (2,191) = 18.619, *P*< 0.000, ɳ^2^ 0.165), Social Support (F (2,191) = 7.57, *P* = 0.001, ɳ^2^ 0.074), Patient Problems (F (2,191) = 3.411, *P* = 0.035, ɳ2 0.035), Sibling Problems (optional scale) (F(2,191) = 5.858, *P* = 0.01, ɳ^2^ 0.058), and Family Beliefs (F (2,191) = 121.649, *P* < 0.000, ɳ^2^ 0.563). PAT scores on the Family Problems and Stress Reactivity subscales did not differ among pathologies (F (2,191) = 1.433, *P* = 0.241, ɳ^2^ 0.015 and F (2,191) = 2.269, *P* = 0.106, ɳ^2^ 0.023, respectively). Post-hoc tests indicated that with respect to Family Structure, those caring for a patient with epilepsy or asthma, displayed higher risk (*P* < 0.05) while Social Support was significantly compromised in the asthma group as compared to the ALL group (*P* < 0.05). A similar pattern was observed for Patient and Sibling Problems. Furthermore, the latter differed also between the epilepsy and asthma group (*P* < 0.05), with families caring for a child with epilepsy displaying a significantly higher risk (*P* < 0.05). Finally, Family beliefs (Fig 1B) were more compromised in families caring for a patient with ALL than in those caring for a child with epilepsy (P < 0.05) or asthma (P < 0.05) (Fig 1B).

**Fig 2:**
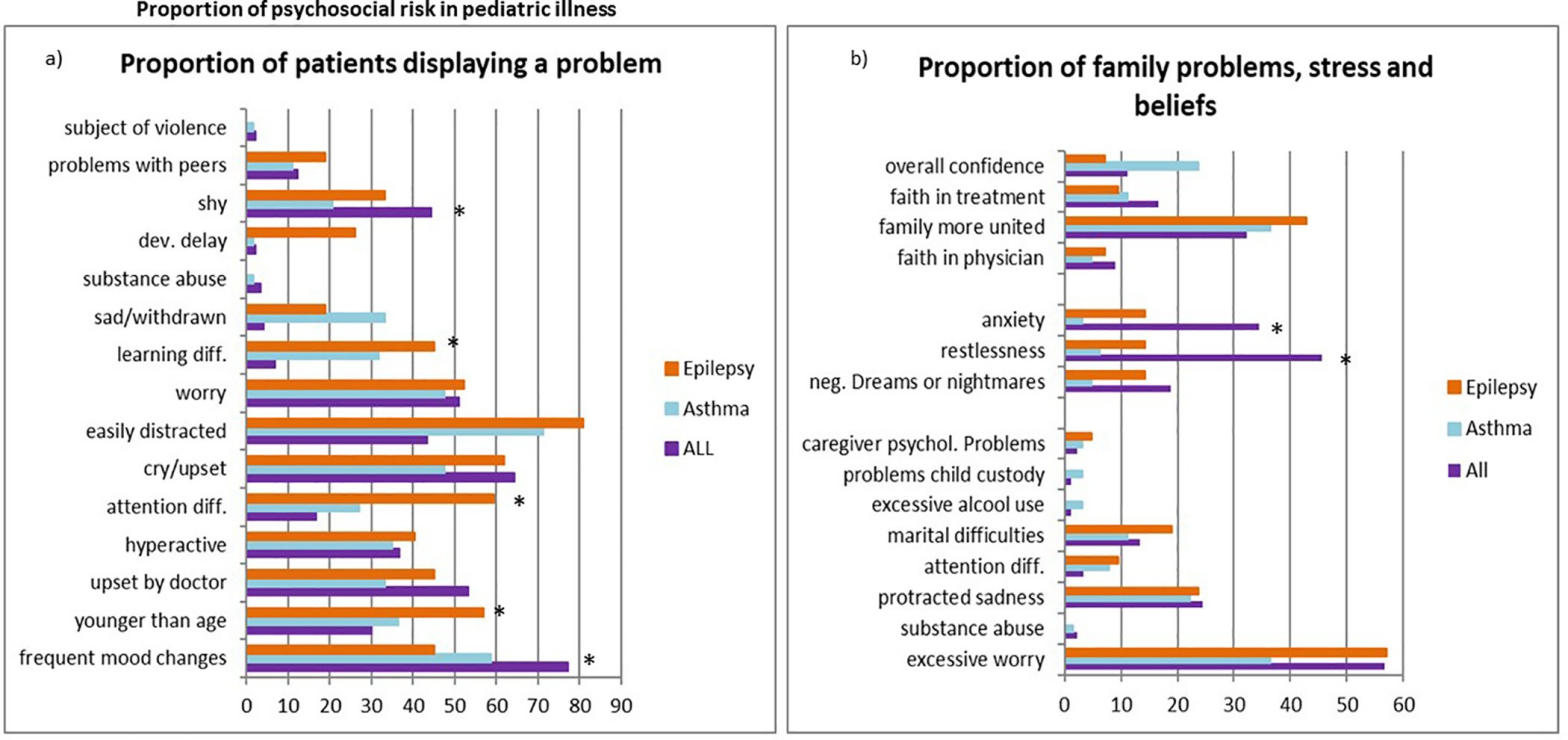
Proportion of psychosocial risk in pediatric illness. a. Proportion of patient problems in children with ALL, epilepsy and asthma; b. Proportion of family problems, stress reactivity, and family beliefs perceived by families caring for a child with ALL, epilepsy, and asthma. Data are expressed as percentages. * indicates p<0.05.

Patient problems constituted an important weight within the total PAT score in patients with ALL, epilepsy and asthma. Children with epilepsy were perceived by their caretakers as worried, overly sensitive, easily distracted, and often displayed attention related difficulties (Fig 2A). Especially, the latter two seem to be characteristic of this patient group; being easily distracted was observed in more than eighty percent of the children with epilepsy which differed significantly from children with ALL (p<0.05), whereas attention different significantly from both patients with ALL (p<0.001) and patients with asthma (p<0.05). Also, children with epilepsy had markedly more parent reported learning difficulties (p<0.001), and a higher incidence of developmental delay (p<0.05) than children with ALL. They also reported more problems with peers, and displayed markedly enhanced behaviors characteristic of children younger than their age. Children with asthma were likewise reported to have attention difficulties, and to be substantially more distracted than children with ALL (p<0.05). Children with asthma were also reported to be sadder than children with ALL while all patient groups reported a high level of worry (almost 50%). Children with a diagnosis of ALL were reported to display more difficulties regarding emotion and were described as suffering more from frequent changes in mood than children with asthma and epilepsy, being worrisome, shy and more afraid of hospital visits (Fig 2A).

The other area of major problems is related to family functioning, that is, family problems, stress and beliefs. Families experience excessive worry ranging from 35% of caregivers of a child with asthma to almost 60%, for those with a child with epilepsy or ALL (p<0.05). Caregivers of a child with ALL reported to be markedly more anxious and restless than parents of children with asthma and epilepsy (p<0,05). A substantial number of families reported to have severe doubts to the unity of their families and feared that their family might fall apart as a result of the strains caused by the disease of their child (Fig 2B). Also, twenty-five percent of parents caring for a child with asthma lacked overall confidence in a positive outcome.

### Network analysis

**Fig 3:**
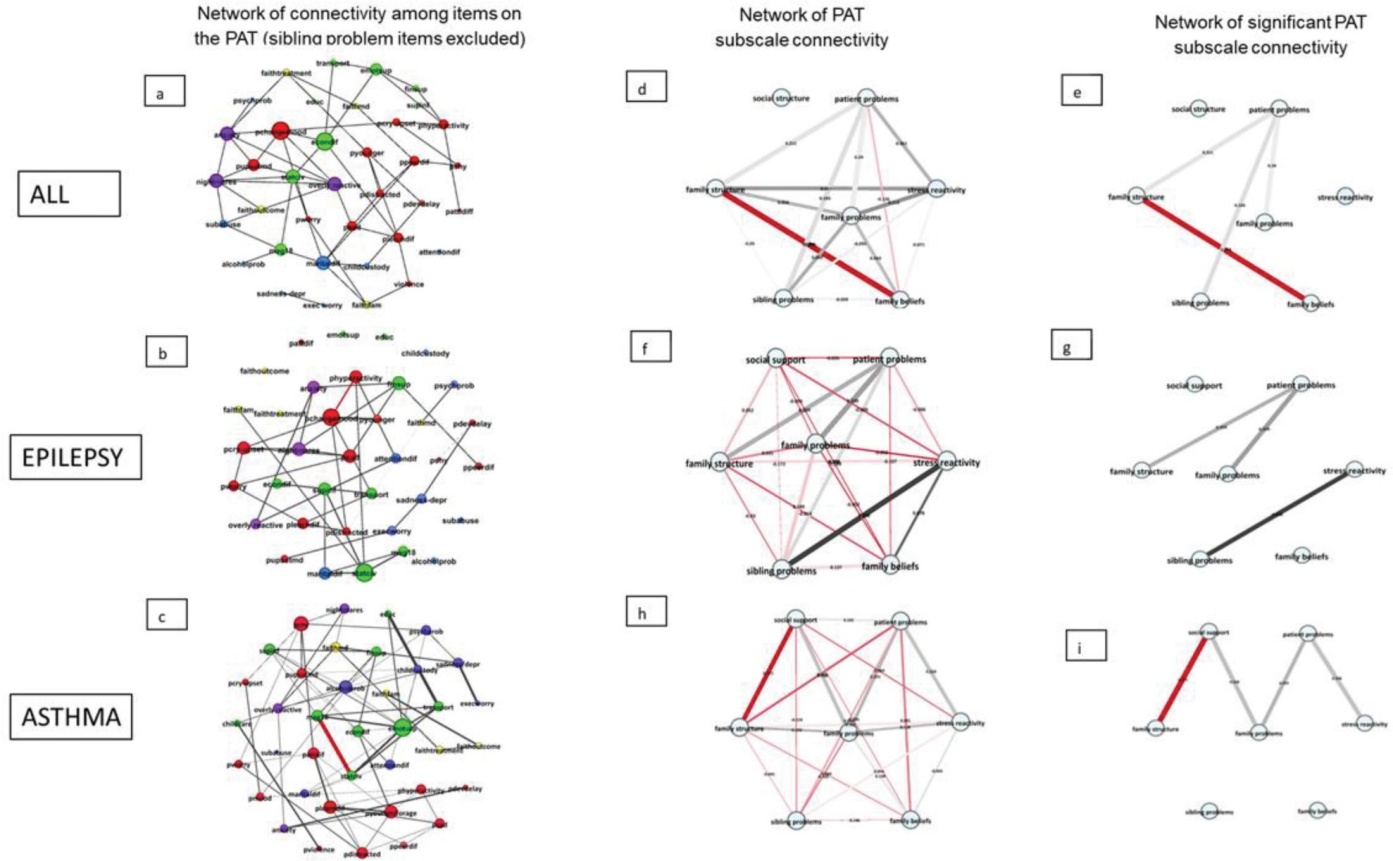
Network of connectivity among items on the PAT. a. Network structure of items on six subscales/domains (sibling problems not included) of the PAT for ALL, epilepsy and asthma. Network structure of items of six subscales/domains (sibling problems not included) of the PAT for ALL (a), epilepsy (b), and asthma (c). Nodes/Items belonging to the same domain have the same color. ● Structure and Resources; ● Social Support; ● Child Problems; ● Adult Problems; ● Family Beliefs; Parent Confidence. The items are plotted using a Fruchterman Reingold algorithm, or spring-plot [91]. Strongly related items are closer to each other while less connected variables are forced more outward and central nodes/variables are placed in the center of the graph. Structure resources and social support: mag18, educ, statciv, finsup, supinf, emotsup, childcare, transport, econdif; child (patient) problems: pchangemood, pyounger, pupsetmd, phyperactivity, pattdiff, pcry-upset, pdistracted, pworry, plearndif. psad, pdevdelay, pshy, ppeerdif, violence; Family (adult) problems: exec worry, subabuse, sadness-depr, attentiondif, maritaldif, alcoholprob, childcustody, psychprob; Stress reactivity: nightmares, overly reactive, anxiety; Family beliefs and attitudes: faithoutcome, faithtreatment, faithfam, faithmd. The thickness of an edge represents the size of the correlation coefficient between two nodes; B. Network structure of (significant) PAT subscales/domains (including sibling problems) for ALL (d,e), epilepsy (f,g) and asthma (h,i) at the first time point. ● Structure and Resources; ● Social Support; ● Child Problems; ● Adult Problems; ● Family Beliefs; ● Parent Confidence. Edges represent correlation coefficients.

Next, a network structure was computed for ALL, epilepsy, and asthma using a covariance matrix of 38 items belonging to six subscales of the PAT (Fig 3). Items on the Sibling Problem scale were not included as this scale was optional. The resulting networks were then analyzed for the importance of each node (item or subscale) within each network, using three measures of centrality: strength (degree centrality), betweenness centrality, and closeness centrality with specific attention for the Patient Problem domain and the Family Related subdomains.

In families caring for a child with ALL (Fig 3A), strong connections existed among items belonging to Family Structure and Family Beliefs while Patient Problems and Family Stress constituted the more central, core problems in the network. We used proportional thresholding to capture only the most robust connections (highest correlations) among the domains of psychosocial function. When considering the domains of psychosocial function that significantly seemed to drive psychosocial risk, two separate highly interconnected forces were detected, (1) those related to the problems of the patient, which negatively influenced family problems and represented an important burden on the structure of the family as well as on other children in the family (siblings) (for ALL and epilepsy where parents reported more on sibling problems than in asthma), and (2) those related to family beliefs and family structure and resources. Importantly, some variables related to the stress reactivity of the caregivers, such as sadness and excessive worry, were placed in the periphery of the network, which means that they are less likely to drive psychosocial risk. Fig 3D displays the fully connected subdomains as well as the domains that ultimately seem to be the most compromised (Fig 3E) in families caring for a patient diagnosed with ALL. Note that, the latter, not fully connected networks serve as a representation of the psychosocial forces most important and are not used as measures to compare the forces among pediatric conditions.

Families of children diagnosed with epilepsy displayed enhanced Patient Problems which were strongly connected to problems in Family Structure and Resources and Family (adult) Problems. As projected in Fig 3B, a second cluster of strongly interrelated domains existed between high Stress Reactivity and Family Beliefs, that is, families displayed reduced confidence regarding the care surrounding their child and future quality of life. However, in these families, social support did not play a role in the psychosocial network of needs and difficulties. In general, the network was represented by two strongly related sets of difficulties, though globally the network was weakly connected (Fig 3F and 3G). Families caring for a child with epilepsy showed an inferior level of psychosocial organization which results in a more fragile, less defined psychosocial structure than observed for instance in families caring for a child with ALL.

Families caring for children with asthma displayed a particular pattern of psychosocial risks. The most compromised domains were Family Structure and the level of perceived Social Support. These domains were strongly connected and occupied a central position in the network (Fig 3C). Also, having few adults in the household and being a single parent together with a lack of emotional support and current economic difficulties weighed especially heavy and seemed to drive the overall psychosocial risk of these families. Within the Family Structure subscale in asthma families, 27% was made up by having few adults in the household (item 1), while other variables in this subscale, such as, education, contributed much less to the subscale score. Also, current economic difficulties (item7/8) weighted heavily on this subscale score (41.2%) and resulted to be a major risk factor. The score on the Social Support subscale was driven primarily by the perceived lack of emotional support and help with childcare (both present in 12.7% of asthma families). Surprisingly, problems strictly related to the patients played a more peripheral role with respect to the psychosocial network and the psychosocial health of these families (Fig 3C). The need for social support and family structure are highly correlated and represent an important area of social risk for these families. Furthermore, these two areas are associated with problems experienced by their child and other family and stress related factors (Fig 3H and Fig 3I). Sibling problems and family beliefs are not part of an integrated network of risk.

Generally, the network is weakly connected. Overall, families caring for patients with severe or chronic pediatric conditions displayed different network metrics (Table 4) with enhanced clustering of items (nodes) in patients with Asthma (Table 4) while the shortest path length was observed in the ALL group.

**Table 4:** Overall network characteristics.

	ALL	Epilepsy	Asthma
Average degree	3.2	2.11	3.64
Network diameter	5	7	7
Graph density	0.094	0.064	0.1
Modularity	0.495	0.624	0.57
Connected components	4	8	2
Average clustering coef.	0.193	0.206	0.405
Average path length	2.742	3.27	3.326

**Fig 4:**
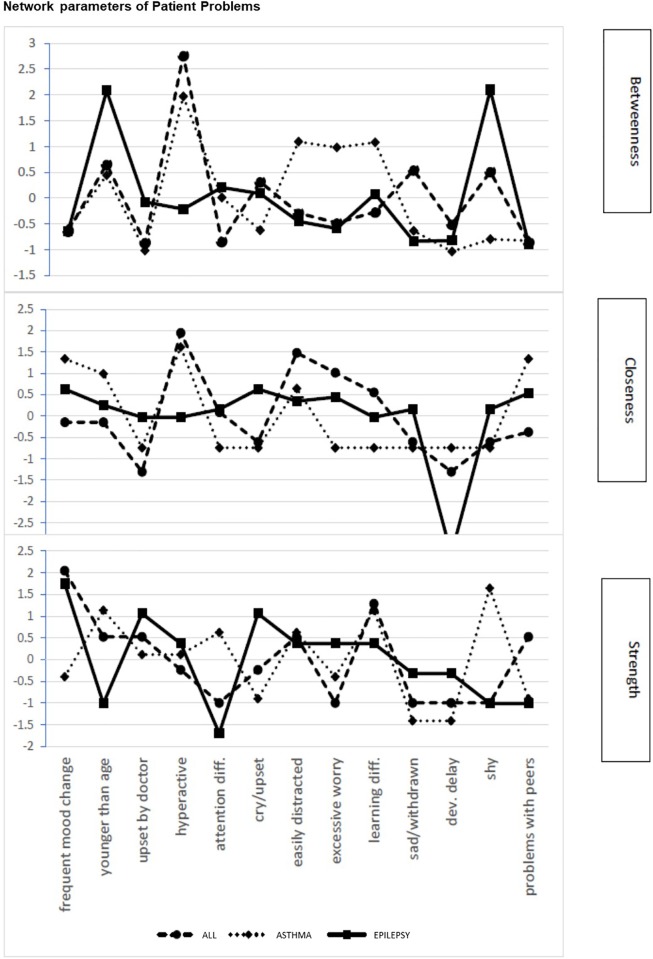
Network parameters of patient problems. Centrality parameters for each item on the Patient Problem subscale of the PAT for each pediatric condition. The upper panel represent betweenness, the middle panel closeness and the lower panel depicts degree centrality for items on the Patient Problem subscale of the PAT and PAT generic. Items are represented in order of appearance on the test.

The (sub)network of problems of children diagnosed with ALL, epilepsy, and asthma differed in betweenness, closeness, and node strength (Fig 4). Higher values reflect greater importance of a symptom or problem within the network. For ALL and asthma the node with the highest betweenness centrality was hyperactivity, which in children with asthma was combined with elevated betweenness centrality for being easily distracted, displaying excessive worry, and having learning problems. In children with epilepsy, highest betweenness was observed for acting younger than their age and being shy. Though these two symptoms have strong betweenness centrality, they are placed more towards the periphery of the network which is further supported by low closeness (Fig 4) and reduced strengths of these symptoms. Node strength was highest for changes in mood in children with ALL as well as in children with epilepsy (Fig 4) and supports the importance and the high frequency of these symptoms (80% in ALL and 50% in epilepsy) as driving forces of patient related risk in both groups. Frequent changes in mood were also present in 60% of children with asthma but played a less central role (Fig 3). Thus, high prevalence of symptoms or problems in a substantial proportion of patients does not always drive the network as indicated by centrality measures.

**Fig 5:**
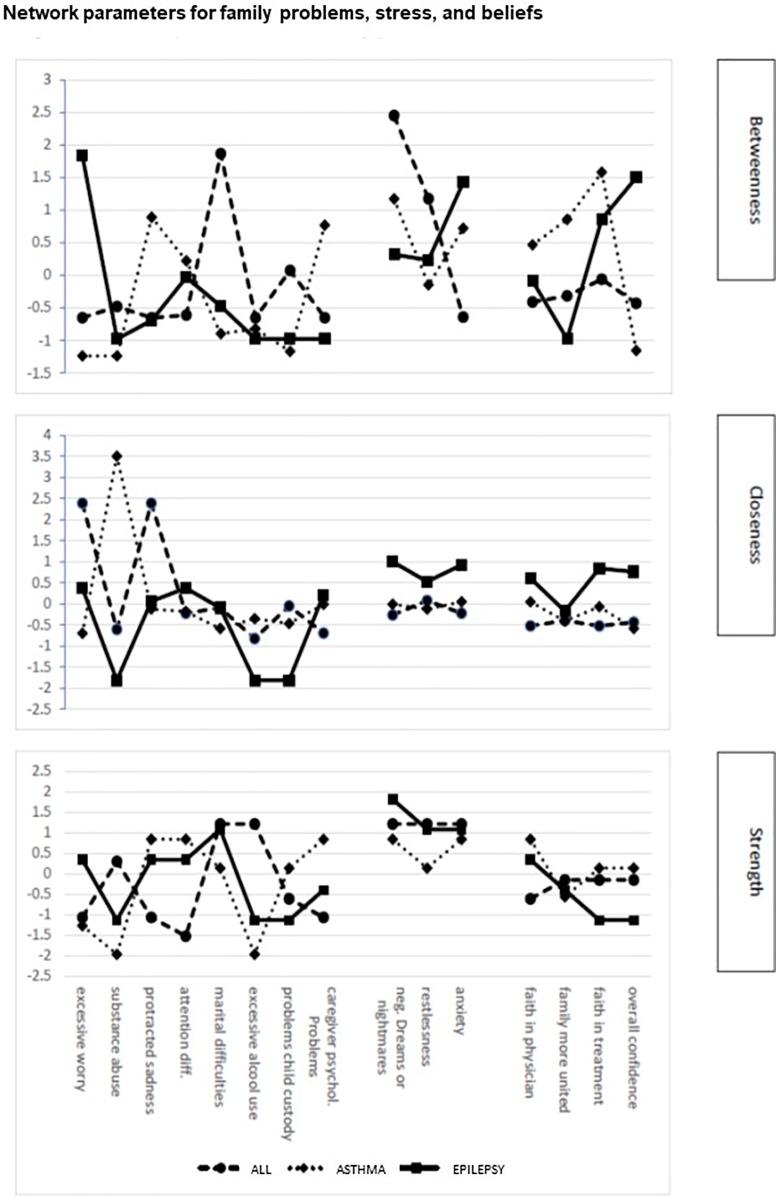
Network parameters for family problems, stress, and beliefs. Centrality parameters for items on the Family Problems, Stress, and Beliefs subscales of the PAT for each pediatric condition. The upper panel represent betweenness, the middle panel closeness and the lower panel depicts degree centrality for items on the Family Problems, Stress, and Beliefs subscales of the PAT and PAT generic. Items are represented in order of appearance on the test.

In families caring for severely ill children family problems, caregiver stress, and beliefs constituted important risk factors (Fig 5). Families caring for a child with ALL displayed excessive worry and protracted sadness with high closeness centrality, though situated in the periphery these symptoms were characterized by low betweenness centrality (Fig 5). This suggests that although highly prevalent (58% and 24% respectively), they do not drive the risk of the Family Problem subscale in this patient group. More central in driving Family problems for this patient group were marital difficulties, showing high betweenness and node strength. Moreover, a similar pattern of centrality was observed with respect to caregiver stress, where restlessness and nightmares showed high betweenness centrality and node strength, and therefore are likely to drive this subscale. Network parameters related to families caring for a child with asthma showed high betweenness centrality and degree centrality (strength) for protracted sadness and caregiver psychological problems as well as nightmares and anxiety (Fig 5). Also, caregivers of this patient group indicate excessive alcohol use as a correlated problem. This suggests that caregiver mood and psychological problems are the driving source of psychosocial risk and that caretakers of patients with asthma have serious personal difficulties in managing their emotions and stress. In addition, an important driving force of psychosocial risk in this group was the perceived lack of social support which was highly associated with low caregiver education, single parenthood, lack of social support and economic difficulties.

Families caring for a child with epilepsy were characterized by less family related problems and more patient associated problems. Betweenness centrality calculated for items on the subscales of family problems, stress reactivity and family beliefs showed that caretakers of children with epilepsy displayed a peak in excessive worry and protracted sadness. However, while highly prevalent, they were less central. More central were caregiver related stress, such as anxiety, restlessness, and negative dreams (Fig 5).

### Data driven cluster analysis

**Fig 6:**
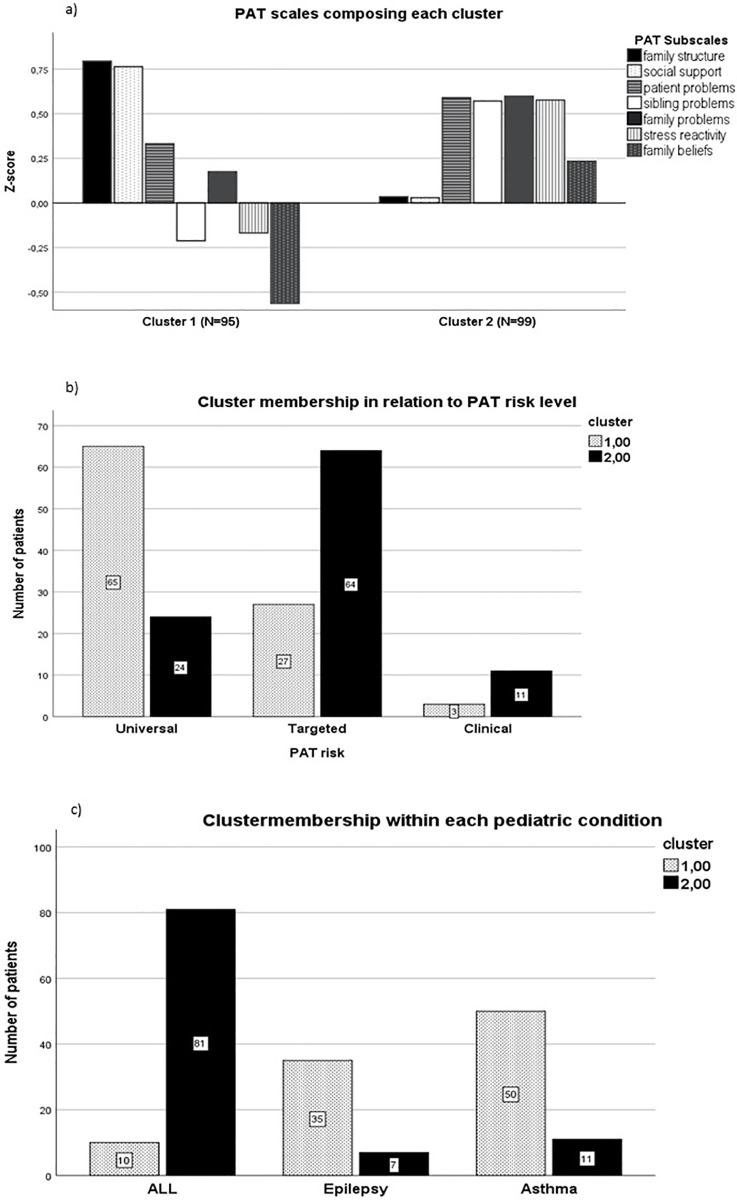
Pattern of PAT domains within cluster 1 and cluster 2. a. Domains composing each cluster displayed different properties. Cluster 1 was composed of substantial problems related to Family Structure and Social Support as well as Patient related problems while Sibling related problems and Family Stress and Beliefs did not negatively affect psychosocial risk of families belonging to this cluster. Families belonging to cluster 2 were characterized by significant problems related to the Patient, Siblings, Family and to Stress Reactivity; b. Cluster membership within each pathology; c. Cluster membership in relation to PAT risk level.

To investigate whether children with severe medical conditions have some psychosocial risk factors in common that are characteristic of families caring for a severe or chronically ill child irrespective of their specific condition, a data driven exploratory cluster analysis was performed [68,99]. Cluster analysis resulted in a two-cluster model in which certain diseases shared problems in specific domains which defined two unique common clusters (Fig 6). Cluster one was characterized by marked presence of problems related to family structure, and social support. In contrast, cluster two was characterized by the almost total absence of problems related to Family Structure and Social Support and by elevated problems regarding Family Beliefs and attitudes toward the care of their ill child (Fig 6A). The three remaining domains, Patient Problems, Family Problems, and Stress Reactivity did not preferentially load on either of the two clusters. Patient Problems and Family Problems embodied a common factor underlying the total PAT, while the domains of Family Structure, Social Support, and Family Beliefs, did not (Fig 6A). Most patients with ALL belonged to cluster 2 (90% 81/90), whereas families of children with epilepsy and or asthma belonged to cluster 1 (83%, 35/42 and 82%, 50/63 respectively) (Fig 6B). Furthermore, families in cluster 1, displayed a low PAT risk level (67.7% 65/96), in contrast, most families belonging to cluster 2 were characterized by an intermediate risk level (65.5% 65/99, which asks for targeted interventions (Fig 6C). Patients in the clinical range did not differ between cluster 1 and 2 (4.2%, 4/96 and 11.11%, 11/99 respectively) (Fig 6C).

## Discussion

The goal of this study was to assess the differences and similarities of psychosocial risk in families caring for a child with ALL, epilepsy or asthma and how to best analyze the dynamic interdependencies among various domains of psychosocial risk. Specifically, the research was designed to answer 4 questions: (1) Does the overall level of psychosocial risk differ among various serious or chronic pediatric conditions? (2) Are domains of psychosocial functioning affected diversely in families caring for a child diagnosed with ALL, epilepsy or asthma? (3) What is the weight of individual psychosocial domains? What psychosocial domain is central to other psychosocial risk factors and is this different for each condition, and alternatively (4) Does a common risk profile exist for families caring for a severely or chronically ill child?

To assess the perceived psychosocial needs and risks of families caring for a child with ALL, epilepsy, and asthma, the PAT 2.0 and the generic version of the PAT were used [78,79]. The latter consisted of the same domains with some questions restated to match the requirements for a more general use without altering the validity and the psychometric structure of the questionnaire. The PAT proved a useful tool in identifying the level of overall psychosocial risk in diverse clinical settings and separated the psychosocial risk of families into three levels reflecting the data from the authors of the scale [75,76]. Overall, domains of psychosocial function are differently affected, and displayed a different weight within the PAT according to the type of pediatric condition. Families caring for a child with ALL displayed the highest PAT total score (1.44) which indicated a need for specific risk related targeted interventions, while those caring for a child with epilepsy or asthma had a score of around 1 (means 1.0 and 0.90 respectively) and mostly required general support (Fig 1A). Surprisingly, for ALL, the proportion of families belonging to distinct risk levels differed with respect to the US sample [88]: a substantial number of families—almost fifty percent—displayed an intermediate risk level while only forty percent of the families belonged to the lowest, universal risk level. Moreover, cluster analyses (cluster 1 was characterized by substantial family structure and social support related problems while cluster 2 typically included more stress reactivity and personal believes related problems) suggest that families who displayed more difficulties regarding stress reactivity and personal believes (cluster 2) belong for seventy percent to the targeted level of psychosocial risk. These are families that need to be monitored: since the greatest difficulties are in the realm of the family, thus interventions in support of the family could help and bring functioning to a universal risk level, while a failure to intervene could put these families at an even higher risk. Most of the families in cluster 2 have endured their child’s ALL, which is known to be a devastating experience for the entire family [100]. Therefore, intervention is necessary taking the whole family into consideration in a timely manner.

One of our main priorities was to understand the dynamic relationship between domains of psychosocial risk for each condition. Relationships within a network can be qualitatively analyzed using graph theory analysis [17,101–103]. Recently, graph theory analysis has been used to study the interrelationships of different symptoms of anxiety, depression or to study cognitive performance in patients with pediatric epilepsy or ADHD [17,18,101,104] where symptoms or domains of cognitive performance are represented as nodes in a network and the associations between the symptoms/nodes as the connections or edges [17,18,89,105]. Also, graph theory has been used, to detect which symptoms, signs and vulnerabilities are central to different clinical diagnoses of psychopathology [93,104]. Given this, we set out to better understand which psychosocial problems [103] and needs are central to specific severe and chronic pediatric conditions and how potential central problems were connected to other difficulties. Overall, we found that different needs exist in families caring for children suffering from different pediatric conditions even though considerable heterogeneity was observed within each condition with respect to the needs of individual families. Families of a child with ALL need substantial assistance and support in dealing with caregiver stress and gaining faith in the efficacy of the treatment protocol of their child, while families with a child with epilepsy need more assistance regarding family structure and resources, and family (adult) related problems. Families caring for a child with asthma also displayed a need for help at the level of family structure but in addition they revealed a need for social support as well. Furthermore, when performing a data-driven cluster analysis, patients fell into one of two clusters. Cluster 1 grouped 80% of the epilepsy and asthma patients while Cluster 2 contained almost 90% of the ALL patients. Comparison between these two clusters partly confirmed the findings of the network and graph analysis in that caring for a child with epilepsy or asthma put similar strains on their families. One explanation for this, may be that epilepsy and asthma are chronic severe diseases but often not life-threatening. In contrast, ALL is considered a potentially life-threatening disease, though for ALL an almost 85% survival rate has been reached [106,107]. Notwithstanding this, the diagnosis of ALL is a family experience: the long, intense, and trying treatment period, characterized by frequent hospitalization, repeated painful interventions, and demanding treatment related side effects, followed by a long period of uncertainty as to the outcome, may cause the family to experience extreme uncontrollable stress, which we found and confirmed to represents the core psychosocial problem of these families [100,108]. Given this, patients and their families are more at risk for the development of for example PTSD, which causes substantial suffering, especially, within the context of regular hospital visits as part of the long-term follow-up of these patients [35]. Substantially less needs were expressed by these families in the domains of Social Support and Family Structure. This may be partially explained by the fact that in pediatric oncology standard guidelines of care are generally accepted, and consequently the sick child and his/her family is surrounded by more structured (psycho) social assistance [108]. Therefore, as opposed to families caring for a child with epilepsy or asthma, family structure, resources and social support, may weigh less, that is, occupy a less central place in the network of psychosocial risk. Families of patients with epilepsy and asthma, in general, receive less structured social assistance while having to manage and organize chronic daily hassles surrounding the care of their child. These, then, may ultimately become a fundamental problem in the relationships, resources, and social support of the caregivers [109–111].

Patient related problems occupy a substantial proportion of the total PAT score occupying the same proportion of psychosocial risk for families with a child with ALL, epilepsy or asthma. This may represents the family's difficulty in coping with the characteristics of the child that are accentuated by the pathology [4,5].

Also, psychosocial risk does not represent a static property of family functioning. Family dynamics change with the age of the cognitive, emotional and social capacities of the patient and interdependencies among these capacities may be influenced by chronological as well as the developmental age of the patient [1,2,20,34]. Especially, developmental change in social and emotional needs from childhood to adolescence may put additional strain on family functioning [43,112]. In this study, patients ranged in age from 5–12 years. Consequently, the behavior and emotional needs of the patient might differ with age as well as their psychosocial ones [113,114]. Often patients displayed a regression and returned to displaying behaviors characteristic of a younger age, such as observed with a higher incidence in children with epilepsy [115]. Given the available data in the literature, when analyzing future time points of this longitudinal study, age and change over time should be included as an important factor.

Several strengths and limitations exist. An important point is that here we used evaluations of items related to various domains of psychosocial function which are based on a multifaceted series of cognitive and emotional considerations as well as on beliefs and attitudes and, thus, represent interdependencies between properties of a much more intricate network. Furthermore, the PAT is not the sole test available to assess psychosocial risk and a broad range of tests and questionnaires exist that measure psychosocial risk. With respect to the pediatric oncology setting, the PAT is certainly one of the most widespread tests currently used, while the use of the generic version is still limited. An important step, then, would be to try and enhance the use the generic version in order to establish a common core of tests that evaluate the same domains of risk in diverse pediatric conditions, which may help to reduce heterogeneity in the results caused by the use of different assessment tools.

Finally, this study is a first effort to analyze the interrelationships among psychosocial risk factors using a network approach [103]. We did not address the impact of variables, such as age of onset or duration of illness, type(s) of treatment, which are all know to play a role in defining psychosocial risk. Further research should consider their role in altering the dynamics of and impact on the psychosocial network of risk and needs underlying each pathology [116]. We should also enhance the number of subjects for each pathology.

The use of graph theory together with other more traditional approaches may provide a new way to explore and comprehend the effect of pediatric severe and chronic conditions [64,116,117], such as, ALL, epilepsy, and asthma on the needs of the families that care for them. Vice versa, this new approach offers a dynamic way of framing the psychosocial risk of these families [118]. More traditional risk assessments lack this dynamic aspect and often fail to establish what forces are typically driving the overall risk distinctive of each pathology. For example, epilepsy and asthma have the same overall PAT score and belong to the same cluster but display diverse interrelationships among the various domains, with different domains constraining the risk of other domains for epilepsy and asthma. In conclusion, in keeping with these results, we should try to develop and implement specific interventions that target the most central, most connected domain, expecting an improvement in the connected domains as well.

## Conclusions

The data suggest that families caring for a child with a severe possibly life-threatening disease such as ALL display a higher level of psychosocial risk than families caring for a child with a chronic disease such as epilepsy and asthma. However, the current study also indicates that each pediatric illness is best described by particular psychosocial and patient related problems that seem to take a central place in driving the overall level of psychosocial risk [3,30,119]. These central problems, then, may represent excellent targets to which to direct therapy, thus, helping the family to manage the problems posed by the illness of their child in a more efficient way. Often problems are inherent to the illness experience and therefore, the goal of intervention is not only to resolve the problem where possible but to assist in preventing future negative consequences so that the negative impact on the long-term quality of life is reduced and the burden of disease is minimized.

However, before we can fully benefit from these results at the clinical level, we must address some important research questions in the future. A first question regards what variables related to psychosocial risk are most salient. In contrast to cross-sectional studies that concentrate their efforts on examining connections among single symptoms, we have focused on the dynamic interaction among variables related to patient problems, such as excessive sadness, frequent changes in mood, and being easily distracted or hyperactive. However, it is yet unclear what variables are most relevant and therefore will be the most suitable and informative to study psychosocial risk in children with severe and chronic pediatric conditions. Second, future research should address the time span during which to measure risk factors, risk related behaviors or emotions. As stated before, needs change during development and currently it is unknown what the appropriate pattern of surveillance is to capture these dynamic developmental changes. Also, future research should include other severe and chronic conditions, such as, type I diabetes, cystic fibrosis or juvenile arthritis. A final important issue for future research is how the analysis of group networks translate to and are predictive of the network of strengths and needs of individual families caring for a child with specific pediatric conditions and how to tailor targeted individualized interventions and predict their effect.

## Supporting information

S1 Fig (JPG)

S2 Fig (JPG)

S3 Fig (TIF)

S1 File (DOCX)
