# Supplementary figures and images for: Psychosocial assessment of families caring for a child with acute lymphoblastic leukemia, epilepsy or asthma: Psychosocial risk as network of interacting symptoms
Source: PLoS One. 2020 Mar 23;15(3):e0230194. doi: 10.1371/journal.pone.0230194 (PMC7089558; doi:10.1371/journal.pone.0230194)

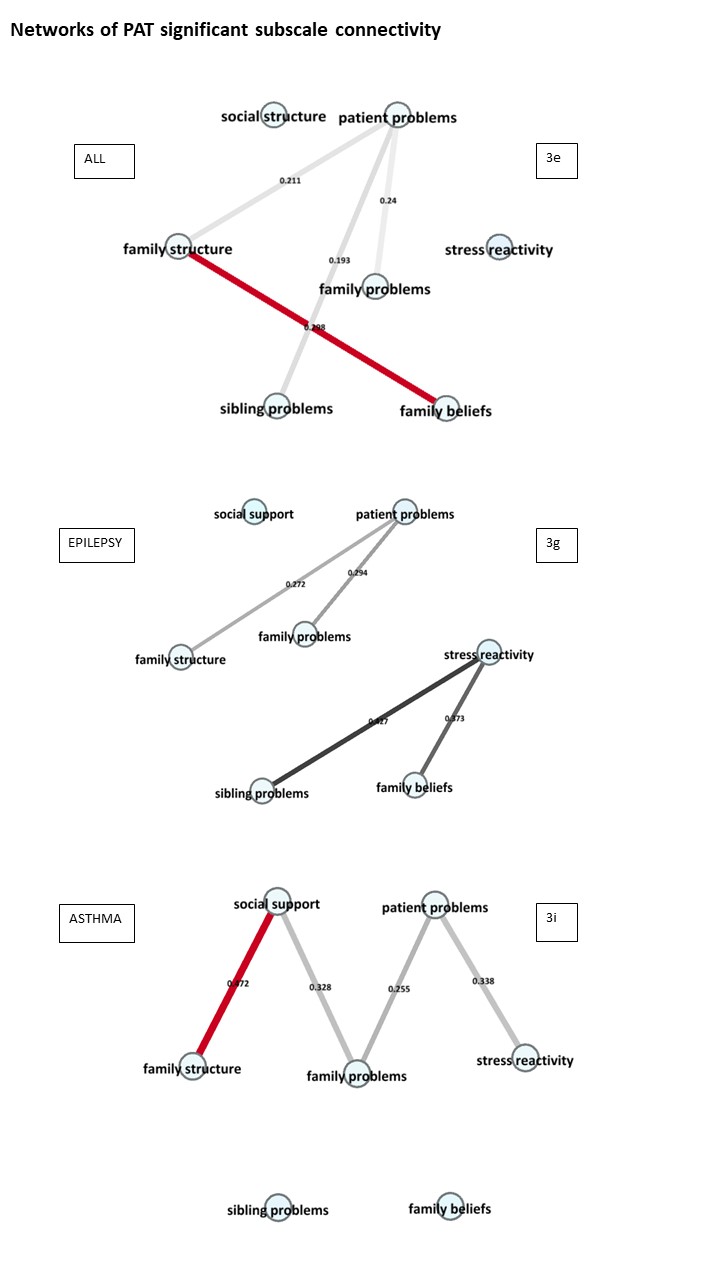

Supplement: S1 Fig — (JPG) [file pone.0230194.s001.jpg]

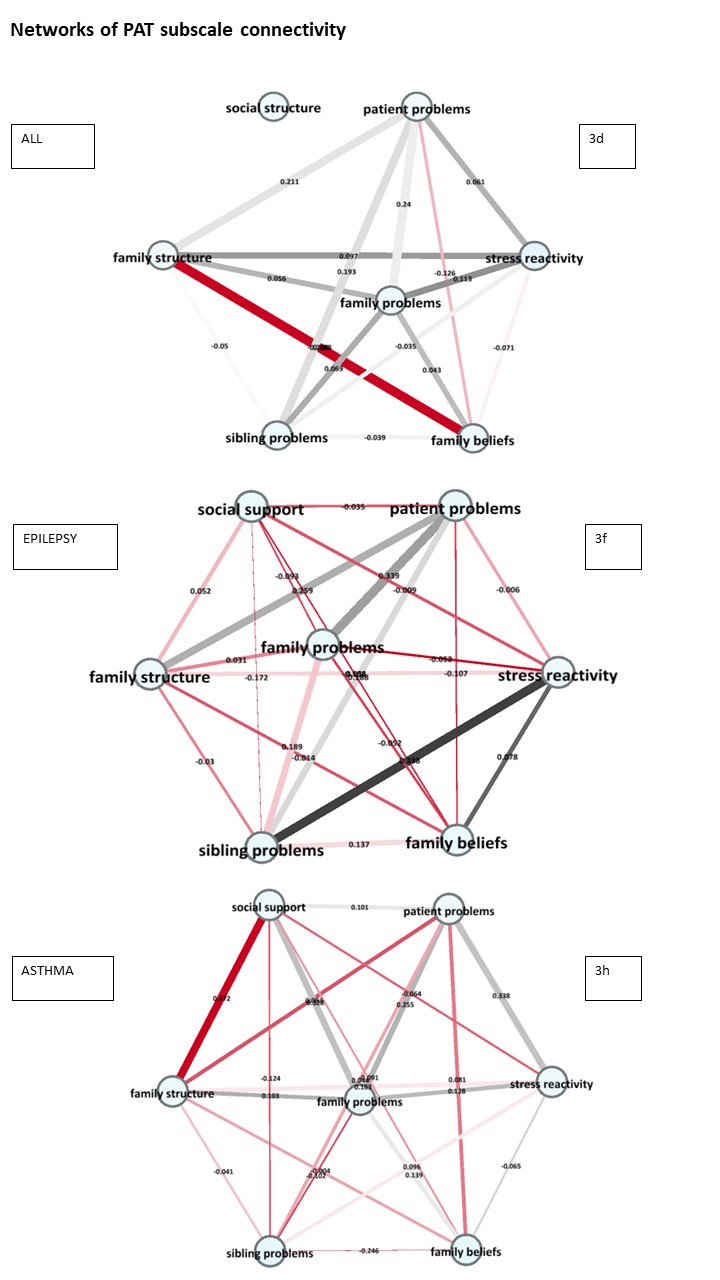

Supplement: S2 Fig — (JPG) [file pone.0230194.s002.jpg]

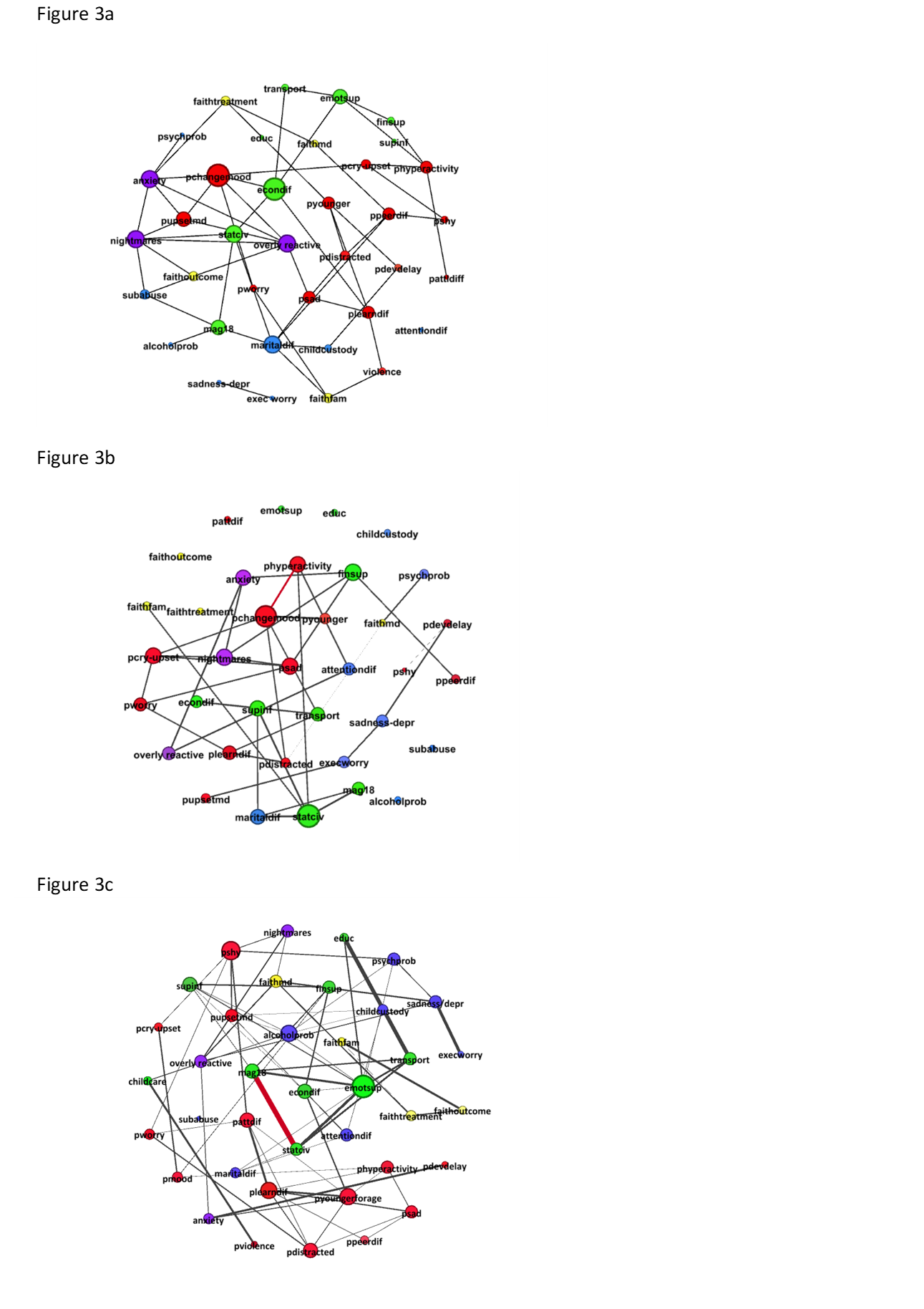

Supplement: S3 Fig — (TIF) [file pone.0230194.s003.tif]
