# Supplementary material for: Psychosocial assessment of families caring for a child with acute lymphoblastic leukemia, epilepsy or asthma: Psychosocial risk as network of interacting symptoms
Source: PLoS One. 2020 Mar 23;15(3):e0230194. doi: 10.1371/journal.pone.0230194 (PMC7089558; doi:10.1371/journal.pone.0230194)
Supplement: S1 File — (DOCX) [file pone.0230194.s004.docx]

The lack of additional information regarding time since diagnosis is an important factor to consider.

We performed an additional analysis and tested the hypothesis that there were different patterns between individuals depending on the years of illness and treatment path. Years of illness differed between patients with epilepsy and patients with asthma (F (1, 103= 17.746, p<.001), mean years of illness for the patients with epilepsy was 3.36 yrs. (SD 2,11 95% CI 2.64-4.07) and for patients diagnosed with asthma 5.9 (SD 3.1, 95% CI 5.07-6.72). The overall mean of years of illness for these pathologies combined was 4.95 (SD 3,1). Interestingly, when testing the effect of type of pathology on PAT subscales scores, with years of illness as factor of covariance, no main effect was observed with respect to pathology and PAT subscales. The only subscale that differed significantly between epilepsy and asthma, was the stress reactivity of the family, which was twice as high in families caring for a child with epilepsy as compared to those caring for a child diagnosed with asthma .120 vs 0.54 respectively). Given these additional analyses, we might conclude that more than years of illness, the medical intensity of antiepileptic treatment leads to heightened stress reactivity as compared to the treatment of children with asthma, which is generally considered to be less invasive. The data suggest the presence of different patterns of stress reactivity which depend more on the treatment path than on years of illness. Also, families of children diagnosed with ALL have the highest relative stress reactivity which seems to be almost entirely related to the intensity of the treatment of acute lymphoblastic leukemia.
